# VIDD or CIADD? The ventilator remains a bona fide culprit

**DOI:** 10.1186/s12871-026-04298-6

**Published:** 2026-10-06

**Authors:** Juan Liu, Xueyan Yuan, Yi Yang

**Affiliations:** 1 Jiangsu Provincial Key Laboratory of Critical Care Medicine, Department of Critical Care Medicine, School of Medicine, Zhongda Hospital, Southeast University, Nanjing, 210009 People’s Republic of China; 2 Department of Critical Care Medicine, Liangjiang Hospital, Nanjing Pukou People’s Hospital, Southeast University, Nanjing, 211800 Jiangsu People’s Republic of China

**Keywords:** Ventilator-induced diaphragm dysfunction, Critical illness-associated diaphragm dysfunction, Mechanical ventilation, Diaphragm-protective ventilation, Brain-dead organ donors

## Abstract

Rosà and colleagues recently proposed that mechanical ventilation (MV) may not be the primary driver of diaphragm dysfunction in critically ill patients, advocating the term “critical illness-associated diaphragm dysfunction” (CIADD) over “ventilator-induced diaphragm dysfunction” (VIDD). While we support distinguishing VIDD from CIADD, we contend that the review’s central conclusion underestimates the causal role of MV itself.

This commentary addresses three questions in turn. First, does MV injure the diaphragm under controlled conditions? Here the evidence is strong: brain-dead donor studies—dismissed by the authors as a “unique patient category”—are a uniquely informative, though not pure, human model of controlled MV exposure, and their dismissal creates an unfalsifiable position. Second, how much does MV contribute to diaphragm dysfunction in critically ill patients? This is considerably less certain: the molecular abnormalities observed in critically ill patients (ubiquitin-proteasome activation, RyR1 calcium leak, mitochondrial oxidative stress) coincide with pathways that MV reliably induces in controlled animal experiments, providing biological plausibility—though not proof—that MV contributes to the molecular phenotype of critical illness. Third, do MV and critical illness interact? We propose that CIADD should serve as an umbrella term encompassing VIDD as a distinct mechanistic subtype—rather than replacing it—analogous to how “pneumonia” subsumes VAP, HAP, and CAP, and we propose three differentiated model types for future research: pure VIDD models, pure CIADD models, and combined models with factorial design.

VIDD and CIADD are mechanistically distinct but interacting entities. Future research should employ models that minimize confounding from critical illness to isolate ventilation-specific injury pathways. Importantly, establishing that VIDD exists is not equivalent to establishing that preventing VIDD is beneficial; clinical trials are needed to demonstrate the feasibility and benefit of diaphragm-protective strategies. Diminishing MV’s role risks undermining the mechanistic foundation of diaphragm-protective ventilation strategies.

Dear Editor,

We read with great interest the comprehensive review by Rosà and colleagues [1], which challenges the long-standing paradigm of ventilator-induced diaphragm dysfunction (VIDD) and proposes the alternative term “critical illness-associated diaphragm dysfunction” (CIADD). We commend the authors for this timely and provocative reappraisal and agree that critical illness per se—sepsis, inflammation, multiorgan failure—is a major independent driver of diaphragm weakness, as evidenced by the high prevalence of dysfunction at ICU admission [2] and its reversibility with sepsis resolution [3]. We also find the concept of muscle hibernation as a potentially protective mechanism insightful [4]. However, we respectfully contend that the review’s central conclusion—that the role of mechanical ventilation (MV) itself is “limited or at least unclear”—conflates two distinct questions: whether MV is the primary cause of dysfunction in ICU patients (debatable) and whether MV can cause diaphragm injury (unequivocally yes). To these we would add a third, and in our view the most clinically consequential, question: whether MV and critical illness interact in producing diaphragm dysfunction—biologically plausible and experimentally important, but largely unresolved. We therefore structure our commentary around these three questions.

## Part 1. Does mechanical ventilation cause diaphragm injury under controlled conditions?

The authors substantially underweight the evidence from brain-dead organ donor studies. Levine and colleagues [5] demonstrated a reduction in diaphragm fiber cross-sectional area of 57% in slow-twitch and 53% in fast-twitch fibers after controlled MV, confirmed by Hussain et al. [6] (myofibrillar dysfunction) and Picard et al. [7] (mitochondrial damage). The authors dismiss these as a “unique patient category” with limited generalizability. Yet this uniqueness is largely their value: brain-dead donors are typically free from overt sepsis, shock, and multiorgan failure, making them a uniquely informative—though not pure—human model of controlled MV exposure. We acknowledge that brain death itself is associated with profound physiological changes, including neuroendocrine, inflammatory, hemodynamic, and metabolic disturbances; these patients therefore approximate, but do not isolate, the effect of MV. Nevertheless, they provide an unusually informative human model for studying controlled MV-related changes in the diaphragm. Dismissing this evidence on grounds of uniqueness creates an unfalsifiable position—as all studies of typical ICU patients are confounded by critical illness itself, there would remain no human population in which MV’s effects could ever be isolated.

Two decades of convergent animal evidence across species consistently demonstrate that controlled MV induces time-dependent diaphragm atrophy, oxidative stress, and contractile dysfunction [8, 9]. Translation gaps reflect model limitations, not absence of causality. Recent mechanistic advances—including mitochondrial fission via DRP1-FIS1 [10], endoplasmic reticulum stress as an independent pathway [11], Foxo3/Txnip-mediated proteolysis [12], and Piezo1- and ITGB1-mediated fibrotic remodeling [13, 14]—further solidify the causal chain from MV to diaphragm injury at the molecular level. Under controlled conditions, the answer to our first question is therefore unequivocally yes.

## Part 2. How much does mechanical ventilation contribute to diaphragm dysfunction in critically ill patients?

The authors note that non-brain-dead critically ill patients exhibit “only” ~25% fiber atrophy, insufficient to fully explain severe weakness [15]. However, they simultaneously acknowledge that sarcomeric protein dysfunction and impaired calcium sensitivity contribute to weakness beyond atrophy [15, 16]. Here we must be precise about what this observation does and does not establish. The molecular abnormalities documented in critically ill patients—ubiquitin-proteasome activation, RyR1 calcium leak, mitochondrial oxidative stress—cannot be assumed to reflect MV-induced injury, because critical illness itself plausibly activates these same pathways. Rather, the demonstration that MV induces precisely these pathways in controlled experimental models—free of critical illness—provides biological plausibility that MV may contribute to the molecular phenotype observed during critical illness. Distinguishing biological plausibility from proven causality is essential: in individual ICU patients, the relative contribution of MV remains uncertain and likely varies with ventilator settings, duration of controlled ventilation, and the intensity of concurrent inflammatory insults.

Recent clinical data provide suggestive, though still indirect, support for MV-specific injury pathways. In a prospective cohort of mechanically ventilated patients, dyssynchronous post-inspiratory diaphragm loading—a ventilator-related exposure—was associated with progressive impairment of diaphragm neuromuscular coupling, independent of inspiratory loading, organ failure severity, sepsis, and neuromuscular blockade; moreover, both diaphragm thinning and thickening over time were associated with declining neuromuscular coupling, linking structural change to functional impairment [17]. Such observations indicate that how the ventilator interacts with the patient’s own respiratory effort matters, but they fall short of quantifying overall MV’s contribution relative to critical illness.

## Part 3. Do mechanical ventilation and critical illness interact?

The most productive path forward is not to debate whether MV or critical illness is “the” culprit, but to explicitly recognize VIDD and CIADD as mechanistically distinct but interacting entities that converge in ICU patients. The interaction may even be bidirectional. In a landmark study, Ebihara and colleagues demonstrated that MV protected the diaphragm against sepsis-induced injury by abrogating an injurious interaction between oxidative and mechanical stresses [18]—evidence that MV and critical illness interact in ways that can be injurious or protective depending on context. This bidirectionality carries a critical methodological implication: future VIDD research should employ models that minimize confounding from critical illness. If the goal is to study MV-induced fibrotic remodeling, introducing sepsis or ARDS into the model creates a confounder that precludes attributing the fibrotic response to MV alone. Conversely, studies of sepsis-induced myopathy must rigorously control and report ventilator settings, as uncontrolled variations may introduce hidden VIDD-related confounders. We therefore propose three model types: (1) pure VIDD models—healthy animals under controlled MV, essential for isolating MV-specific mechanisms; (2) pure CIADD models—sepsis/inflammation without MV, for dissecting inflammatory pathways; and (3) combined models with factorial design, for dissecting the direction and magnitude of MV–critical illness interactions.

## Personalization, not abandonment

We agree that diaphragm-protective targets should not be pursued at the expense of lung protection or cognitive preservation. However, the absence of definitive clinical evidence for load-induced myotrauma does not constitute evidence of absence—particularly given the formidable ethical barriers to such evidence. The nuanced approach is personalization: controlled diaphragm rest may be appropriate during acute sepsis—consistent with the observation that MV can protect the septic diaphragm [18]—while active diaphragm-protective strategies may benefit patients without major inflammatory confounders. The RESCUE-3 trial [19] showed that temporary transvenous diaphragm neurostimulation improved weaning success (70% vs. 61%, HR 1.34). We acknowledge, however, that the intervention was brief and delivered during the weaning phase in patients already receiving assisted ventilation; as such, RESCUE-3 demonstrates the feasibility and short-term benefit of augmenting diaphragm activity during weaning, but it does not address whether maintaining diaphragm activity throughout controlled MV prevents VIDD. More generally, asserting that VIDD exists is not the same as asserting that preventing VIDD is known to be beneficial; clinical trials are needed to demonstrate the feasibility and patient-centered benefit of diaphragm-protective strategies.

In conclusion, we support distinguishing VIDD from CIADD—the authors’ most important conceptual contribution. However, CIADD should serve as an umbrella term encompassing VIDD as a distinct mechanistic subtype, rather than replacing it. Just as “pneumonia” subsumes VAP, HAP, and CAP to guide targeted therapy, CIADD should encompass VIDD, sepsis-induced dysfunction, and critical illness myopathy to advance precision medicine. Our commentary has limitations: it is a perspective grounded in observational and preclinical evidence, and we cannot quantify the relative contributions of MV and critical illness in individual patients. Whether preventing VIDD translates into improved patient-centered outcomes therefore remains to be demonstrated in adequately powered clinical trials of diaphragm-protective strategies. Nonetheless, diminishing MV’s role risks undermining the mechanistic foundation that underpins diaphragm-protective ventilation—a foundation built on convergent evidence from human and animal studies that deserves continued, differentiated investigation.

## Data Availability

No datasets were generated or analysed during the current study.

## Abbreviations

ARDS: Acute respiratory distress syndrome
CAP: Community-acquired pneumonia
CIADD: Critical illness-associated diaphragm dysfunction
DRP1: Dynamin-related protein 1
ER: Endoplasmic reticulum
FIS1: Mitochondrial fission 1 protein
Foxo3: Forkhead box O3
HAP: Hospital-acquired pneumonia
HR: Hazard ratio
ICU: Intensive care unit
ITGB1: Integrin beta-1
MV: Mechanical ventilation
Piezo1: Piezo-type mechanosensitive ion channel component 1
RyR1: Ryanodine receptor type 1
Txnip: Thioredoxin-interacting protein
VAP: Ventilator-associated pneumonia
VIDD: Ventilator-induced diaphragm dysfunction
